# Supplementary material for: The utility of infectious disease modelling in informing decisions for outbreak response: A scoping review
Source: PLOS Glob Public Health. 2025 Sep 2;5(9):e0005120. doi: 10.1371/journal.pgph.0005120 (PMC12404446; doi:10.1371/journal.pgph.0005120)
Supplement: S1 File — Fig A. Screenshot of final search strategy and query on Ovid. S1 File Table A. Rationales for the search strategy and query on Ovid. S1 File Fig B. Number of included articles by year of publication. S1 File Fig C. Number of included articles by considered target population in the model. S1 File Fig D. Number of included articles by considered disease or pathogen in the model. (PDF) [file pgph.0005120.s002.pdf]

S1 Text

Methods: Details of the search strategy

As mentioned in the main text, the literature search was conducted via Ovid. The final search from June 5 2024 is depicted in S1 Text Fig A. The rationales behind the components of the total search string are described in S1 Text Table A.

S1 Text Fig A. Screenshot of final search strategy and query on Ovid.

▲	Searches	Results
1	((model* or forecast* or simulat* or projection* or scenario*) adj9 (outbreak* or epidemi* or pandemic*)).ti,ab,hw,kf.	75585
2	"infectious disease model*".ti.	259
3	("disease model*" and (Sars-Cov-2 or COVID-19 or measles or Zika or diphtheria or mpox or HIV or influenza or ebola)).ti.	131
4	1 or 2 or 3	75791
5	(decision* or policy or policymaking or policies or public health or priorities or planning or guideline*).ti,ab,hw,kf.	5767567
6	4 and 5	23517
7	(Sars-Cov-2 or COVID-19 or measles or Zika or diphtheria or mpox or HIV or influenza or ebola).ti,ab,hw,kf.	2207110
8	6 and 7	13279
9	(assess* or evaluat* or utility or analy* or useful* or effect*).ti,ab,hw,kf.	43593239
10	8 and 9	11093
11	limit 10 to yr="2019 -Current"	8644
12	(protocol* or editorial* or correspondence* or "letter to the editor" or "conference abstract*").ti,ab.	1876844
13	(cost-effectiv* or benefit* or vaccin*).ti,ab.	3541217
14	11 not (12 or 13)	6127
15	limit 14 to "remove preprint records"	5924
16	remove duplicates from 15	4007

S1 Text Table A. Rationales for the search strategy and query on Ovid.

**Lines in search strategy**	**Rationale**
1-4	Include all publications that are related to disease models to consider the outbreak model inclusion criterion
5-6	Require a connection to policy to consider the outbreak model inclusion criterion
7-8	Fulfill the inclusion criterion considering certain diseases
9-10	Fulfill policy evaluation inclusion criterion

11	Fulfill time inclusion criterion
12+15	Fulfill peer-review inclusion criterion
13	Fulfill pharmaceutical intervention and cost-effectiveness exclusion criteria
14	(See the above the rows)
16	Remove duplicates

Results: Additional characteristics of the included articles

The majority of the studies included were published in public health journals with the most number of articles being from the 'British Medical Journal' followed by the journal 'Epidemics'.

Within the considered time frame from 2019-2024, most publications were from 2021 (S1 Text Fig B). This is in line with the fact that most articles considered the COVID-19 pandemic.

S1 Text Fig B. Number of included articles by year of publication.

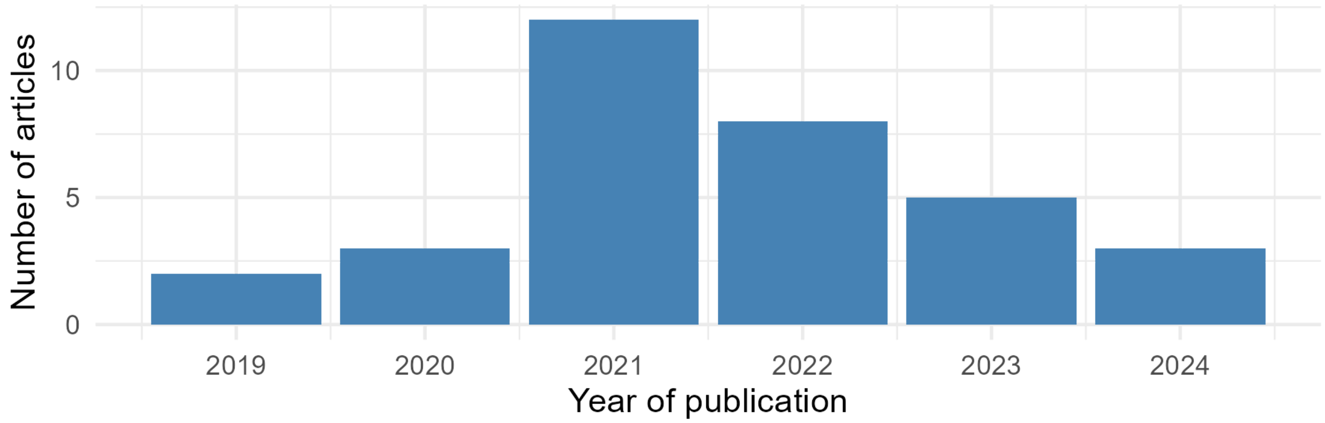

Most included publications are from the first years of the COVID-19 pandemic.

The modelling efforts that are described in the included articles consider a variety of target populations within a geographical area (S1 Text Fig C). However, most of them consider the total population of a country or of a region in a country.

S1 Text Fig C. Number of included articles by considered target population in the model.

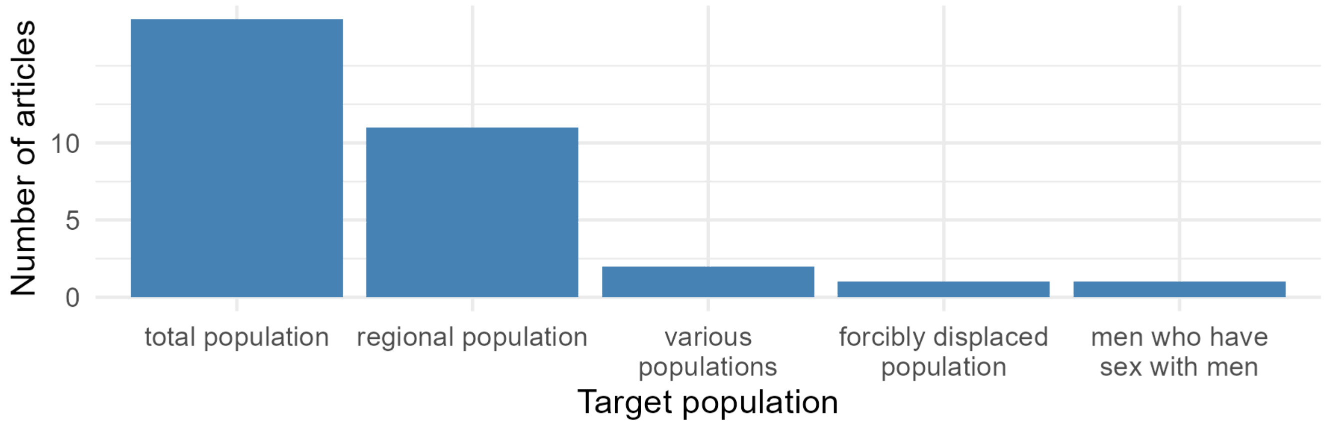

In their modelling efforts, included articles mostly consider the total population of a country or of a region within.

Even though the search went beyond COVID-19 related articles, most included articles consider COVID-19 (S1 Text Fig D).

S1 Text Fig D. Number of included articles by considered disease or pathogen in the model.

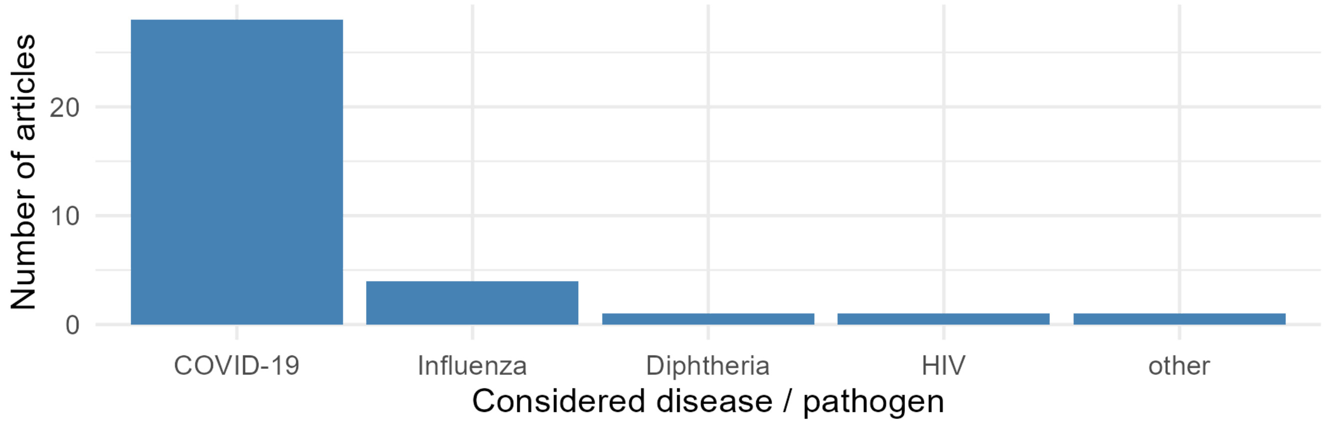

Included articles mostly consider COVID-19. Note that some articles consider multiple diseases/pathogens.
