# Supplementary material for: The utility of infectious disease modelling in informing decisions for outbreak response: A scoping review
Source: PLOS Glob Public Health. 2025 Sep 2;5(9):e0005120. doi: 10.1371/journal.pgph.0005120 (PMC12404446; doi:10.1371/journal.pgph.0005120)
Supplement: Attachment — Submitted filename: rebuttal letter.pdf [file pgph.0005120.s005.pdf]

Dear reviewers, dear Charin Modchang, Ph.D.,

Thank you very much for your helpful feedback. In the following, we address your points one by one.
As we think that it has raised the quality of our work, we would like to thank you for your ideas and suggestions in our Acknowledgements if you allow (p. 27, l. 483f):

> "Lastly, we thank the reviewers for their constructive comments and suggestions, which have improved this review substantially."

Please note that the referenced page and line numbers refer to the clean version of our manuscript.

Sincerely,
Emil Iftekhar
(on behalf of all coauthors)

Reviewer #1:

This is an important work that presents the use of math modelling into the policy despite its presence since decades. I have no comments - authors presented very well with strong methodology with proper interpretations and conclusion.

> Thank you very much!

Reviewer #2:

The author have conducted a scoping review "to assess how infectious disease modelling inform policy, focusing on challenges and facilitators of translating modelling insights into actionable policies.

1. As mentioned by the authors, this manuscript is quite similar to ref [9], and the authors attempt to distinguish findings between the two manuscripts in the discussion.

> We hope our replies and changes in reaction to your comment 4 and comment 2 of reviewer 3 have clarified this further.

2. A point of contention in phrasing "modelling to policy" that is mentioned several times. A more correct phrasing would be modelling to decision-support as many of these models supporting resource allocation are not really geared into action that that results in a policy revision or new policy being written.

> Thank you. This is a good point. Hence, we have reframed it to "modelling-for-decisions" and replaced "policy" by "decisions" in the title and in many other instances throughout the manuscript. We have replaced "to" by "for" to signal that it is not necessarily a linear translation process but can be a collaborative, iterative process as found in this review.

3. What is not clear from the review is the impact made… i.e. who listened to what evidence and how that was consumed?

> Due to the nature of the included articles, i.e. mostly self-reported reflections and not objective assessments, the impact of modelling for decisions is indeed not clear in the articles. We have described what is reported about

- who listened to evidence in the decision maker subsection of the Results (p. 14),
- what evidence has been presented in the policy objectives and role of modelling subsection of the Results (p. 13),
- and how that was consumed in the approaches to policy interaction subsection of the Results (p. 15).

We hope that our explanations in response to comment 3 of reviewer 3 clarify the limitations of the included articles of this review.

4. Given that the majority of LMIC articles in the came from [16,17] – a consortium originated and led by Oxford University, to what extent does the review represent global experience?

You are right in questioning that! We have clarified it further in the Results section under "Summary of extracted" data (p. 11, l. 175f) by adding the sentence:

> "While the CoMo Consortium informed decision makers in LMICs, most modellers in the consortium were from HICs."

We also updated the description of Fig 2 (p. 11) to

> "**Fig 2. While many countries worldwide are technically represented, most of the experiences described in this review involve High Income Countries (HICs).** World map highlighting the countries that were considered in the included articles. Although all continents are represented, the majority of articles consider HICs. Note that some articles consider multiple countries; two of these articles account for most of the countries of the Low-and-Middle Income Countries (LMICs) in this figure [16, 17]. Both refer to a modelling consortium of mainly HIC modellers informing LMIC decision makers."

Lastly, we clarified it in our Limitations section of the Discussion (p. 25, l. 442-446):

> "While these described modelling efforts informing LMIC policy makers, they mainly involved HIC modellers [16, 17]. This points to a possible lack of local modelling expertise used in LMIC policy and also means that modelling efforts involving LMIC modellers are underrepresented in our review. However, the comparison between our findings and those of Owek et al.'s focusing on LMICs shows many similar insights, emphasizing the robustness of our results [9]:"

5. Important themes are highlighted in the review, but they are done univariately. It would have been useful to link the barriers with other characteristics of the candidate studies. E.g. Was it mostly ABM models that considered infrastructural issues as a barrier etc. Is that possible with this sample size? This could be presented diagrammatically.

This is a great idea. We had initially also looked at such relationships without success. Even after checking again now, we are not able to find any pattern in our extraction matrix. We have made this investigation transparent in our Results (p. 11, l. 167-170):

> "The summary of the extracted data is presented along the categories of the data extraction. No clear relationships or patterns between the different categories could be identified, e.g. whether a specific modelling approach was more or less prone to a certain barrier. Hence, the categories are summarized independently."

We also discuss this further in our Discussion (p. 23, l. 399-403):

> "In this review, we presented the facilitators, barriers and all other characteristics of modelling-for-decisions pathways independently for each other and were not able to identify relationships and dependencies between those characteristics. This can be explained by the diversity of existing modelling-for-decisions pathways and experiences; the available evidence and level of detail gathered from a scoping review may not be large enough to allow for a more detailed analysis."

And in p. 26, l. 457-460:

> "However, we did not find any studies that provided systematic assessments of the utility or evaluations of the pathways for modelling-informed decisions. This also contributed to the fact we were not able to find clear relationships between characteristics of the pathway."

(Previously already existing text is shown in grey here.)

6. Given that the study did not achieve its aim of assessing the "utility of mathematical modelling in informing policy during infectious disease outbreaks", is there a study design that renders this possible accounting for the nature of decision-making?

Unfortunately, we have not been able to find any work on this specifically. Our team actually wants to fill this gap that we have identified through our review. As a first step towards such a study design, we are currently conducting a study to develop evaluation tools for the modelling-for-decisions pathway. (See our research protocol here: Iftekhar, E. (2025). Policy Impact and Process Evaluation of Advanced Analytics to inform Public Health Decision-Making. https://doi.org/10.17605/OSF.IO/J9QXV) We included this citation in our conclusion and outlook section (p. 26, l. 466ff):

> "These evaluations could involve prospectively defining criteria in collaboration with decision-makers that would enable retrospective evaluation once the modelling work has completed, but also more general reflections of modellers on model accuracy, utility and the relationship between the two [62]."

(Previously already existing text is shown in grey here.)

Reviewer #3:

This manuscript addresses an important and timely topic: how infectious disease modelling informs public health policy during outbreaks. The review is methodologically sound, well-structured, and offers valuable insights into facilitators and barriers along the modelling-to-policy pathway. However, several areas could be clarified or expanded to strengthen the manuscript's utility for both researchers and decision-makers.

Thank you for this detailed, thought-through feedback. We hope that our clarifications and expansions in the following are sufficient to address it.

Major Points

1. Definition of Mathematical Modelling

The manuscript would benefit from a clearer definition of "mathematical modelling," especially for readers outside the field. The current discussion leans heavily on traditional infectious disease models (e.g., differential equations, agent-based, stochastic models), but increasingly, governments and stakeholders are engaging with machine learning (ML)-based approaches. While neural networks are briefly mentioned, there is no discussion of how these fit within the modelling-to-policy context. Additionally, much of the ML-related literature is not indexed in the databases searched (e.g., dblp.org, IEEE, NeurIPS, ICML), and this limitation should be noted—particularly as these models are now being shared with and used by decision-makers. This doesn't require changing the scope of the review but should be clarified in the framing and limitations.

This is a good point. We have extended our definition in the introduction on p. 3, l. 49-54:

> "Mathematical modelling has emerged as an important tool in this context, often referred to as 'infectious disease modelling' (e.g. [2]). It supports outbreak response in multiple ways: Modellers can for example estimate key epidemiological parameters, forecast outbreak size and assess the potential impact of different

control measures (e.g. [3, 4]). They do so by describing epidemiological data and phenomena by equations or explicit rules, and performing simulations or statistical analyses based on them."

(Previously already existing text is shown in grey here.)

We have also included a paragraph to our Limitations section (p. 24, l. 415-420):

"Another subset of literature that we have not actively considered is on machine learning-based approaches to inform decisions. Although some of the included literature considers machine learning-based models, such as Bayesian inference models or even neural network-based models, our search strategy and choice of databases aimed to find literature that considers more explicit modelling approaches. However, it is important to note that more black-box-type machine learning approaches are seen as promising directions in infectious disease modelling and public health policy [46] [47]."

46. Panteli D, Adib K, Buttigieg S, Goiana-da-Silva F, Ladewig K, Azzopardi-Muscat N, et al. Artificial intelligence in public health: promises, challenges, and an agenda for policy makers and public health institutions. The Lancet Public Health. 2025;10(5):e428-e32.

47. Kraemer MUG, Tsui JLH, Chang SY, Lytras S, Khurana MP, Vanderslott S, et al. Artificial intelligence for modelling infectious disease epidemics. Nature. 2025;638(8051):623-35.

2. Clarification of Owek et al. [9]

The review refers to Owek et al. as a scoping review, but the study also involved interviews, workshops, and mapping exercises. These methods enabled the authors to explore interpersonal, political, and structural barriers in greater depth. Clarifying the methodological differences will help readers understand why certain issues (e.g., rivalry between modellers and resistance from officials) appeared in their findings but not in this review.

This is a great idea. We have added these clarifications to our Limitations section (p. 25, l. 437ff):

"Owek et al. have conducted a multi-method study on this topic, involving interviews, workshops, mapping exercises and a scoping review. Their review concentrated on COVID-19 in LMICs and used a different search strategy, including different search terms [9]."

And l. 450f:

"These barriers were not found in our review and may have been identified from the other activities of their multi-method study."

(Previously already existing text is shown in grey here.)

3. Limits of Self-Reported Reflections

The review should more clearly acknowledge that most findings are based on reflections from modellers about their own work. As such, interpersonal or political tensions, institutional power dynamics, or failed engagements may not be reported or may be selectively described. While the review notes this in passing, more explicit discussion of what cannot be captured through this method would improve the interpretation of results. This should be reflected both in the Limitations and in the framing of the Results and Discussion sections.

Thank you for the feedback that it is not clear enough yet. We further clarified it in the beginning of the Results with a sentence on p. 11, l. 165f:

"31 of the 33 included articles are descriptions and reflections by modellers about their own modelling efforts where they highlight aspects they deemed relevant for

the utility of their work in informing policy for outbreak response. Only in two articles, the perspectives of decision makers are portrayed [14, 15]. In the following, we summarise these subjective reflections."

And of the Discussion on p. 21, l. 355-360:

"In this scoping review, we aimed to get an overview of the infectious disease modelling literature that assesses its utility in informing decisions for outbreak response. We included 33 peer-reviewed articles, none of which systematically assessed the utility of their modelling effort for decisions. Instead, authors more subjectively reflected on their modelling effort(s) and highlighted key aspects they deemed relevant for utility. Hence, this review does not present objective utility assessments but summarises those subjective reflections."

We also expanded the Limitations of the Discussion (p. 24, l. 421-427):

"Further, our review is likely affected by publication biases: Some of them are related to the fact that the included articles are subjective assessments, mostly reflecting the perspectives of the authors of modelling studies on what they considered important for the utility of the process. Hence, this review can only capture what authors were willing to share. Authors may prefer not to report or only selectively describe attempts to inform decisions that were perceived as particularly challenging or unsuccessful. In particular, these challenges may involve interpersonal or political tensions, or institutional power dynamics. This might also mean that only more manageable barriers were represented in our analysis."

To streamline the Discussion, accounting for these changes, we have shifted the comparison to general evidence-informed policy research to the first part of the Discussion (p. 22, l. 368-389):

"…These frameworks not only facilitated the rapid generation of scientific evidence but also promoted open communication and trust between decision-makers and modellers, which was critical for the co-creation of decision-relevant evidence during the pandemic.

This is in line with general findings about policy that is informed by any kind of scientific evidence. To systematically evaluate evidence-informed policy making, various models and frameworks have been developed to organize existing knowledge in policy development [45]. These frameworks, commonly utilized in health policy and systems research, include the Ottawa Model of Research Use (OMRU) [46], The Framework for Research Dissemination and Utilization (FRDU) [47] and The Canadian Health Services Research Foundation model (CHSRF) [48], among others. Many of the facilitators of the modelling-for-decisions pathway identified in this review closely align with the guiding principles of those frameworks for evidence-informed policy making. For example, both the OMRU and FRDU frameworks emphasize the importance of involving multiple stakeholders at various levels of the healthcare system in the knowledge-to-policy translation, while the CHSRF advocates for the creation of communication channels among researchers, decision makers, and research funders to foster trust and facilitate the mutual exchange of knowledge. Such collaborative approaches, particularly for modelling-for-decisions pathways, are also praised by others [3, 49, 50], which further highlights the importance of bringing scientists and decision makers together for modelling initiatives. In total, this emphasises the partial transferability of general evidence-informed policy research to modelling-evidence-informed policy research.

Our review also identified several barriers that were thought to be hindering the utility of modelling for the decision-making process."

(Previously already existing text is shown in grey here.)

4. Modelling Choices and Policy Uptake

The review does a good job categorizing model types, but it misses the opportunity to explore how the choice of model influenced clarity, trust, or uptake. For example, ensemble models (e.g., FluSight Network) increased credibility when projections aligned, while more complex models may have been harder to communicate when data was limited. A comparative analysis—even if qualitative—of how deterministic vs. stochastic, statistical vs. mechanistic, or ML vs. traditional frameworks influenced accessibility, interpretability, or actionability would be a valuable addition.

This is a sub-analysis we have made related to comment 5 of reviewer 2. As written in our reply to that comment, we have not been able to find any pattern in our data and have clarified this in our Results and Discussion.

5. Data Quality and Availability

Data-related challenges are mentioned but discussed in general terms. Given how critical this issue is, a deeper discussion would benefit both modelers and stakeholders. Consider expanding on common data limitations (e.g., lack of disaggregated or behavioral data, legal constraints, reporting delays) and how these impacted model design or policy relevance. This would offer practical insights for those working to improve modelling processes and data systems.

Thank you for this suggestion. We have rewritten and extended our paragraph in the facilitators and barriers subsection of the Results (p. 20, l. 326-340):

> "13 of the included studies highlighted barriers in data infrastructure. In general, the study of decision makers in North-Carolina mentions hesitancies in the use of modelling due to "concerns about the quality of data used in models" [15]. Delayed and inconsistent epidemiological data reporting emerges as the most pervasive challenge across modelling efforts [19, 21, 25, 28, 34, 37, 39, 46]. A lack of granularity of data hindered addressing subpopulation-specific questions [15, 25, 42]. Sherratt et al. show that "biased representations of subpopulations in each data source" cause differences in transmission estimates, highlighting the need for decision-makers to be made aware of source populations of used data [42]. The examples, where detailed data was available, were in turn able to provide more nuanced results [19, 42, 45]. Access to data was sometimes enabled through partnerships: collaboration with field staff in Bangladesh [21], with ministries in Austria [28], United Kingdom [45] and Costa Rica [26], and other policy makers [17]. However, the use of modelling was also highlighted as a tool to correct for bad data quality [17, 19, 39], e.g. through hierarchical modelling to account for missing data [19] and by modelling data delays [21]. Another key type of data that was mentioned to be lacking was behavioral data [15, 45]. It made it difficult to accurately model human behavior. An extended description of data-related challenges is described by Steinberg et al. [25]."

(Previously already existing text is shown in grey here.)

6. Stakeholder Involvement

The review notes stakeholder engagement in 15 studies but doesn't break down the depth or phase of this involvement. Were stakeholders involved in framing the initial questions, selecting interventions, or only reviewing final outputs? Were they funders, implementers, or external reviewers? A clearer typology (e.g., co-creation, iterative collaboration, one-time consultation) would help readers understand how engagement influenced policy relevance and uptake.

Thank you very much for the questions and suggestions. We like your suggested typology and have used it to classify the articles in a new table. We have rewritten parts of the stakeholder input subsection to clarify this information (p. 16, l. 262-282):

> "Stakeholder input refers to the contributions, feedback, and interactions of individuals or groups with an interest in the modelling process or those who may be impacted by its outcomes. This may happen through one-time consultations, iterative collaborations or even continuous co-creation of the modelling process. Iterative collaboration may involve processes, where modellers regularly receive feedback on results to further refine their models. Co-creation goes beyond that, e.g. through joint discussions about the models throughout the process.
>
> 15 out of 33 studies benefited from stakeholder input (Table 5). In 10 of these studies, stakeholders were involved via collaborative processes, whereas the information provided in the articles did not always allow for a clear classification into iterative collaboration or co-creation. Input was mostly provided via implementers, including policy makers and clinicians, and external non-modelling experts, such as socioeconomic experts and public health professionals. Only the Pandemic Influenza Outbreak Research Modelling project in Canada reported collaboration with an affected population, in this case the local indigenous community [23]. In general, the input was incorporated in various aspects of the process such as during the selection of variables, setting assumptions, and identifying critical questions to address in the models.

Table 5. Stakeholder input can be included through different processes and by various types of stakeholders.

**Article**	**Process of stakeholder input**	**Type of stakeholder(s)**
George et al., 2023 [38]	One-time consultation (at the end)	Implementers
Irvine et al., 2021 [37]	Iterative collaboration	Implementers
Shea et al., 2023 [32]	One-time consultation (in between)	Research coordination facilitator
Martin et al., 2020 [22]	Co-creation	Implementers
Tariq et al., 2021 [23]	Iterative collaboration	Affected population
Warde et al., 2021 [28]	Iterative collaboration	Implementers
Locey et al., 2020 [43]	Co-creation / iterative collaboration	Implementers
Loo et al., 2024 [18]	Co-creation / iterative collaboration	Implementers
McCaw and Plank, 2023 [44]	Iterative collaboration	External non-modelling experts, press
Howerton et al., 2023 [39]	One-time consultation (in the beginning of every modelling round)	External non-modelling experts, implementers
Keeling et al., 2022 [42]	One-time consultation (in the beginning)	External non-modelling experts

Hendy et al., 2021 [40]	Iterative collaboration	External non-modelling experts, implementer
Adib et al., 2021 [16]	One-time consultation (in the beginning)	External non-modelling experts
Aguas et al., 2020 [17]	Co-creation	External non-modelling experts
Abubakar et al., 2021 [14]	Co-creation / iterative collaboration	External non-modelling experts

The information in the articles did not always make clear whether the process of stakeholder input was one of iterative collaboration or co-creation.

Collaborative efforts…"

(Previously already existing text is shown in grey here.)

7. COVID-19 vs. Non-COVID-19 Comparison

Given that most studies focused on COVID-19, it would be helpful to compare these to the non-COVID-19 studies to assess whether modelling uptake, stakeholder dynamics, or data issues differed. Policymaker expectations and urgency were different during the pandemic, which may have shaped how modelling was used.

Indeed, we had also looked into such differences. Unfortunately, the sample size was too small to observe any clear differences between COVID-19 and non-COVID-19 studies, as we write on p. 12, l. 188f:

"Our data extraction yielded no key differences between modelling efforts about COVID-19 and other diseases."

8. Inclusion of Stakeholders in the Review Itself

Consider discussing whether stakeholders (e.g., policy decision-makers, public health authorities) were involved in designing or interpreting this review. Including such voices might have added valuable perspectives on what makes modelling useful in practice.

This is true. We have added a sentence to the paragraph in the Limitations about our modelling perspective bias (p. 24, l. 428f):

"Additionally, fully understanding modelling-for-decisions pathways requires more perspectives of decision makers and other stakeholders."

And in our Conclusion (p. 26, l. 460-463):

"As we mostly found reflections by modellers, we may have missed important perspectives of decision makers and other stakeholders. While a more diverse authorship of this review may have influenced the study protocol and interpretation of results, it cannot make up for this lack of perspectives in the literature."

Minor changes:

The word mpox should be lower case if not at the start of a sentence.

Thank you. We have corrected it accordingly on pages 3, 4 and 6.

Check that all citations are incorporated into endnote ({Finger, 2019 #4019}).

Thank you for noticing that! We have corrected it on pages 12 and 13.

“modelling-to-policy pathway” is used throughout but once appears as “modelling to policy process”

Thank you for noting this inconsistency. We have corrected this in response to reviewer 2’s second comment.
